# Effects of Resting, Consecutive, Long-Duration Water Immersions on Neuromuscular Endurance in Well-Trained Males

**DOI:** 10.3389/fphys.2018.00977

**Published:** 2018-07-27

**Authors:** Christopher M. Myers, Jeong-Su Kim, Megan Musilli, Kevin McCully, John P. Florian

**Affiliations:** ^1^Department of Nutrition, Food and Exercise Sciences, Florida State University, Tallahassee, FL, United States; ^2^United States Navy Experimental Diving Unit, Panama City Beach, FL, United States; ^3^Department of Kinesiology, University of Georgia, Athens, GA, United States

**Keywords:** water immersion, neuromuscular endurance, electromyography, near-infrared spectroscopy, muscle oxidative capacity

## Abstract

**Purpose:** This study examined the effects of repeated long-duration water immersions (WI)s at 1.35 atmospheres absolute (ATA) on neuromuscular endurance performance. We hypothesized that, following 5 days of consecutive, resting, long-duration WIs, neuromuscular endurance performance would decrease.

**Methods:** Fifteen well-trained, male subjects completed five consecutive 6-h resting WIs with 18-h surface intervals during the dive week while breathing compressed air at 1.35 ATA. Skeletal muscle endurance performance was assessed before and after each WI, and 24 and 72 h after the final WI. Muscular endurance assessments included 40% maximum handgrip endurance (MHE) and 50-repetition maximal isokinetic knee extensions. Near infrared spectroscopy was used to measure muscle oxidative capacity of the vastus lateralis and localized muscle tissue oxygenation of the vastus lateralis and flexor carpi radialis. Simultaneously, brachioradialis neuromuscular activation was measured by surface electromyography.

**Results:** A 24.9% increase (*p* = 0.04) in the muscle oxidative capacity rate constant (*k*) occurred on WI 4 compared to baseline. No changes occurred in 40% MHE time to exhaustion or rate of fatigue or total work performed for the 50-repetition maximal isokinetic knee extension. The first quartile of deoxygenated hemoglobin concentration showed a 6 and 35% increase on WIs 3 and 5 (*p* = 0.026) with second quartile increases of 9 and 32% on WIs 3 and 5 (*p* = 0.049) during the 40% MHE testing when compared to WI 1.

**Conclusion:** Our specific WI protocol resulted in no change to muscular endurance and oxygen kinetics in load bearing and non-load bearing muscles.

## Introduction

Water immersion, complete submersion of the body in water, alters physiological function during and after water egress (Florian et al., 2013a,b, 2014; Myers et al., 2017). Skeletal neuromuscular performance alterations following repeated long-duration WIs, such as those experienced by military, commercial, and technical divers, with exposures longer than 6 h at 1.35 ATA, are not well understood. Data from previous research suggest that these types of exposures are comparable to those seen in microgravity environments (Myers et al., 2017). Short and long-term exposures to microgravity analogs are known to alter skeletal neuromuscular performance in weight and non-weight bearing muscle groups, also known as load- and non-load bearing muscles. The longer the exposure to the WI unloading environment, the greater the alteration to performance, neuromuscular signaling, and muscle microanatomy (Desaphy et al., 2005; Ogneva et al., 2011; Cotter et al., 2015).

The potential underlying mechanisms of change in skeletal muscle performance appear to begin with alterations to signal conductance (Desaphy et al., 2001, 2005). After 3 days of hindlimb unloading in rats, an increased sodium conductance in soleus Type I muscle fibers occurred matching the conductance of Type II fibers (Desaphy et al., 2005). Furthermore, after 3 weeks of hindlimb unloading, messenger ribonucleic acid for sodium and calcium channels increased (Desaphy et al., 2001, 2005). Human models that used 7-day dry immersion methodology, such as Koryak (1999, 2002), demonstrated decrements in action potential duration by 18.8%, and amplitude by 14.6%, during skeletal muscle maximal voluntary contractions. These changes were accompanied by a 33% reduction in maximal voluntary contraction force and a 2.8% decrease in surface area. These variations appear to occur in both load and non-load bearing muscle groups. However, the alterations are more significant in load-bearing muscles while under unloading conditions (Adams et al., 1994; De Boer et al., 2008). De Boer et al. (2008), showed an 8% decrease in VL thickness and no change to biceps brachii thickness after 5 weeks of bed rest. These analog studies simulate the changes in neuromuscular strength seen in long-term exposure to microgravity.

The results between microgravity analog studies versus actual exposure to microgravity on neuromuscular endurance appear to be mixed. Kamiya et al. (2000) conducted a 30% maximum handgrip endurance (MHE) test before and after 14-days of head-down bed rest. Time-to-fatigue was unchanged with increased post- head-down bed rest muscle sympathetic nerve activity. Similarly, Florian et al. (2013b, 2014) reported no change in 40% MHE time-to-fatigue after five consecutive resting, long-duration WIs. Contrary to these results, Fu et al. (2002) reported a significant decrease in MHE time-to-fatigue with increased muscle sympathetic nerve activity after 12–13 days of space flight. These studies demonstrated how the microgravity environment would affect non-load bearing muscles in ways that might be dependent on the length of exposure to the environment. In all cases, localized tissue oxygenation was not measured to investigate whether or not oxygen availability is a limiting factor to muscular endurance performance when decrements to MHE occurred.

Our previous studies using long-duration WIs have demonstrated decrements to muscular strength after 3 days with full recovery by 72-h after the last exposure (Myers et al., 2017). However, little is still known about the effects of these types of exposures on load- versus non-load bearing muscular endurance as only handgrip endurance was investigated (Florian et al., 2013b, 2014). Moreover, the previous research did not measure skeletal muscle tissue oxygenation and neuromuscular activation. The endurance handgrip test for the non-load bearing muscles has been performed in our previous studies (Florian et al., 2013b, 2014). Therefore, this follow-up assessment will allow us to make comparisons to our previous findings after long duration WIs. The 50-repetition isokinetic knee extension has been shown to be a stable analog to measure neuromuscular endurance for load-bearing muscles (Kawahara et al., 2008). These exercise protocols were chosen to assess the reproducibility of previous protocols and to compare these measurements from our previous WI results (Florian et al., 2013b, 2014). Furthermore, these exercise protocols provide reliable and stable movements to measure localized tissue oxygenation and neuromuscular activation. As such, the aim of this investigation was to examine the effects of WIs on neuromuscular endurance performance of load-bearing versus non-load bearing muscles, as well as tissue oxygenation. To address the gap in research into skeletal muscle endurance performance after resting, consecutive, long-duration WIs at 1.35 ATA, we assessed MHE and 50-repetition isokinetic knee extension performance, neuromuscular activation, and localized tissue oxygenation. We hypothesized that over a 5-day period of consecutive, resting, long-duration WIs there would be a decrease to neuromuscular endurance performance and tissue oxygenation with the quadriceps muscle, but not with the forearm flexors.

## Materials and Methods

### Subjects

Fifteen well-trained male divers ($\dot{V}$*O*_2max_ = 51.5 ± 6.7 mL/kg/min) with an average age of 31 ± 6 years old and 7 ± 5 years (mean ± SEM) of diving experience participated in this study (**Table 1**). Before starting the study, each subject underwent a health screening that included a general physical, electrocardiogram, complete metabolic panel, lipid profile, and blood pressure measurements. Exclusion criteria included any known pulmonary, cardiovascular, or metabolic diseases, alcoholism, asthma, and tobacco use. Subjects did not consume any prescription, over-the-counter drugs, or supplements for the duration of the study unless authorized by on-staff medical doctor. A medical doctor, trained in diving physiology, reviewed all subjects’ medical histories. Approval for the study was given by the Institutional Review Boards for the Navy Experimental Diving Unit and Florida State University. Prior to starting the study, each subject provided written informed consent, and all procedures conformed to the Declaration of Helsinki.

**Table 1:** Subject characteristics.

Subject characteristics	
Age (yr)	29.3 ± 1.2
Height (cm)	180.6 ± 2.3
Weight (kg)	88.2 ± 10.3
Body mass index (kg/m^2^)	27.6 ± 2.3
VO_2max_ (mL/kg/min)	51.5 ± 6.7

*Across subject (n = 15) mean (± SEM) characteristics.*

### Study Design

#### Overview

Each subject abstained from food and drink, except water, for 2 h prior to each laboratory visit. Additionally, they were prohibited from performing any fatiguing exercise 48 h prior to any scheduled testing. During all testing sessions, subjects wore running shorts and T-shirts. Subjects completed 5 consecutive days of 6-h resting WIs with 18-h surface intervals with follow-up physiological testing occurring 24 and 72 h after the fifth WI, termed the dive week. Physiological testing during the dive week occurred immediately before, and after, each WI. All testing was performed in a climate-controlled laboratory (21.1–22.8°C). Each subject consumed a small, standardized breakfast consisting of 69% carbohydrates, 19% fats, and 12% proteins (350 kcal) and 236.5 mL of orange juice after the pre-WI physiological testing. Before entering the test pool, each subject voided his bladder, was weighed, and donned a condom catheter (Florian et al., 2013b). Subjects breathed compressed air at 1.35 ATA while resting in a reclined position and did not perform any type of physical activity during each 6-h WI. After the WI, each subject removed his catheter, voided his bladder, dried off, and was weighed (Faes et al., 2013; Florian et al., 2013b). A medical doctor specializing in diving physiology was on site during each dive, and evaluated each subject as needed after each WI for potential side effects. Post-WI physiological testing began immediately after weighing and evaluation by the medical doctor. No adverse effects were reported.

### Familiarization and Baseline Testing

All subjects visited the laboratory three times prior to the first test day and completed these sessions no later than 72 h prior to the first WI. During the first two visits, each subject familiarized himself with the physiological testing protocols. Dynamometer configuration and sensor-placement measurements were recorded during the first visit to ensure replication during subsequent visits.

### Water Immersion

**FIGURE 1:**
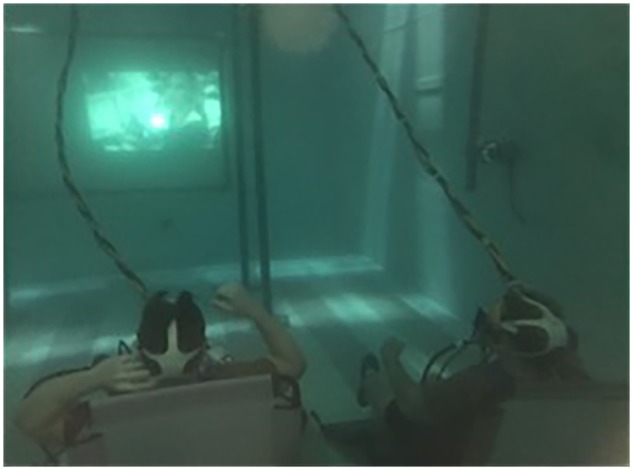
Photo of resting water immersions. Subjects sat in reclining chairs in a thermoneutral (31.7–32.7°C) water tank (15-ft) for 6 h. Atmospheric air was fed from the surface via hoses to the subjects.

The WI protocol occurred as outlined in Florian et al. (2013b). Subjects sat in reclining chairs in a thermoneutral (31.7–32.7°C) water tank (15-ft) for 6 h (**Figure 1**). At the 3-h mark, subjects surfaced up to mid-chest for a 10-min break for a standardized lunch. The meal consisted 64% carbohydrates, 24% fat, and 12% protein (475 Cal) and 500 mL of liquid (Florian et al., 2013b). Once finished, each subject returned to the bottom of the tank to complete the WI (Florian et al., 2013b).

### Data Collection

#### Biodex System Pro 4

Subjects performed the 50-repetition maximal isokinetic knee extension endurance protocol on a Biodex System 4 Pro (Biodex Medical Systems, Shirley, NY, United States). The chair angle was maintained at 85° for upper and lower body performance testing (Harbo et al., 2012; Coggan et al., 2014). Subjects remained seated and strapped to the Biodex during all testing and recovery periods. Subjects did not grasp the Biodex chair or the straps nor were given any verbal encouragement during the exercise protocols. All Biodex attachment measurements and chair configuration measurements were determined during familiarization testing and used throughout the entire study.

#### Endurance Handgrip Equipment

All MHE measurements were taken with the Baseline BIMS HG dynamometer (White Plains, NY, United States) connected to a National Instruments NI USB 6210 module (Austin, TX, United States) and Panasonic Toughbook (Newark, NJ, United States) with National Instruments Labview collection software (Austin, TX, United States) (Florian et al., 2013b). While performing the MHE protocol, the subject’s elbow was flexed at a 90° angle, wrist straight, and thumb orientated toward the ceiling while gripping the dynamometer. Each subject performed the MHE protocol while sitting on the Biodex. Subjects were not given any type of encouragement during the testing.

#### Surface Electromyography (SEMG)

One SEMG (Delsys Trigno Wireless EMG systems, Boston, MA, United States) sensor recorded neuromuscular activity during the MHE protocol. Each subject’s skin was prepared with an alcohol pad and shaved to ensure proper electrical conductance. The sensor was attached to the belly of the brachioradialis using specialized, double-adhesive tape (Delsys Trigno adhesive, Boston, MA, United States). All SEMG signals were pre-amplified (×100), amplified (×2), band-pass filtered (10–1000 Hz), and sampled at 2000 Hz with Trigno EMGWorks software (version 4.1.7, Boston, MA, United States). Roberts and Gabaldón (2008) Raw SEMG data was converted by using Fourier Transformation root mean squared (RMS) script via EMG Works Analysis software (Delsys, Boston, MA, United States). The raw SEMG amplitude data for all previously mentioned exercise protocols were normalized to baseline and used for statistical analysis (Powers and Howley, 2011).

#### Near-Infrared Spectroscopy (NIRS)

This study used a 2-wavelength, portable, continuous-wave NIRS system (Portamon, Artinis, Medical System, Zetten, Netherlands). The system utilized the modified Beer–Lambert and spatially resolved spectroscopy methods to measure changes in deoxyhemoglobin (HHb) and oxyhemoglobin (HbO_2_) concentrations at 760 and 850 nm wavelengths (Ufland, 2012; Ufland et al., 2012). Due to an overlap in wavelengths, myoglobin concentrations could not be separated from hemoglobin concentrations. Each NIRS device had three fixed optode sets capable of penetrating 35 mm into the target tissue. A Portamon sensor was placed on the belly of the VL and medial forearm flexor muscles, primarily the flexor carpi radialis (Ufland, 2012; Ufland et al., 2012). Each placement was measured, marked with permanent marker, and recorded during baseline testing to ensure placement consistency before each test. The probe was attached to the skin using black kinesio-tape to prevent corruption of the signal from ambient light from the surrounding environment (Ufland, 2012; Ufland et al., 2012). Skinfold thickness at the sight of probe application was taken using Lange skinfold calipers (Cambridge Scientific Industries, Cambridge, MA, United States) (Shibuya and Tanaka, 2003; Felici et al., 2009; Ufland et al., 2012). The calculated thickness of the skin and subcutaneous tissue was less than half the distance between the probe and measured target (∼17 mm) (Shibuya and Tanaka, 2003; Felici et al., 2009; Ufland et al., 2012). All NIRS testing was acquired at 10 Hz via a laptop with Windows 8 and recorded for post-exercise analysis. HHb concentration and TSI measurements were recorded and analyzed via statistical analysis.

### Experimental Procedures

#### MHE Protocol

MHE testing occurred pre- and post-WI for the dive week, and 24-hr and 72-hr post-WI 5. Subjects held 40 ± 2% of his baseline MHG strength until fatigue. A digital real-time output visually dictated whether the subject needed to tighten or lessen his grip to maintain the proper force output. The subject’s time was recorded using a stopwatch and began when the subjects’ grip reached 40 ± 2% as indicated by the software. The protocol ended once the subject could no longer maintain the required force or voluntarily discontinued the test (Florian et al., 2013b). The total grip time (seconds) was recorded and analyzed. All subjects performed testing with their right hand.

Simultaneously, real-time SEMG and NIRS data were recorded. Brachioradialis SEMG data were transformed via RMS and normalized to baseline values. Since time-to-fatigue differed from subject to subject and individual testing sessions, the SEMG normalized amplitude data, HHb values, and TSI values were averaged into quartiles using the customized script for MatLab version R2016A (The Mathworks, Natick, MA, United States). The results of the quartile analysis were used for statistical analysis (Kutsuzawa et al., 2009).

#### 50-Repetition Maximal Isokinetic Knee Extension Protocol

The 50-repetition maximal isokinetic knee extension occurred pre- and post-WI 1 and 3, pre-WI 5, and 24-hr post-WI 5. The exercise protocol was limited to these days as to minimize artificial fatigue in the subjects. Before beginning of the test, each subject was briefed on the exercise protocol and instructed to give maximal effort. Each subject performed one set of 50 repetitions of maximal isokinetic knee extension at an angular rate of 180°/s concentric and 300°/s eccentric (Larsson et al., 2003; De Ste Croix et al., 2009; Coggan et al., 2014) with his right leg. Only one set of the 50-repetition maximal isokinetic knee extension exercise was performed during certain testing sessions as to minimize the effects of fatigue and skeletal muscle microtrauma (De Ste Croix et al., 2009). The peak torque (Newton-meters) for repetitions 2–4, 24–26, and 48–50 and total work were recorded. Upon completion of the fatigue protocol, the subject was permitted to rest in place until all NIRS readings returned to pre-exercise levels.

The slope of decline in the concentric force outputs was calculated. Furthermore, the decline in force output (slope) acted as a control to measure the maximal output for each individual. The total work and the rate of fatigue were statistically analyzed (De Ste Croix et al., 2009; Coggan et al., 2014). For the NIRS data, peak and steady-state HHb and TSI values were measured and then consolidated via a customized script for MatLab version R2016A. The relative changes for HHb and TSI were calculated and used for statistical analysis (Kutsuzawa et al., 2009).

#### Muscle Oxidative Capacity

The PortaMon NIRS sensor placement was used to assess muscle oxidative capacity as outlined in the Ryan et al. (2012). A key assumption made about this technique is the signal changes are in hemoglobin are proportional to mitochondrial oxygen consumption (Ryan et al., 2013). Three consecutive tests were conducted on the VL with 3 min of rest between each test. A pneumatic cuff was strapped to the most proximal portion of the right leg. The cuff was attached to a pneumatic pump capable of inflating the cuff to suprasystolic pressure equal to 300 mmHg. The pneumatic pump was capable of inflating and deflating the cuff within 1 s.

**FIGURE 2:**
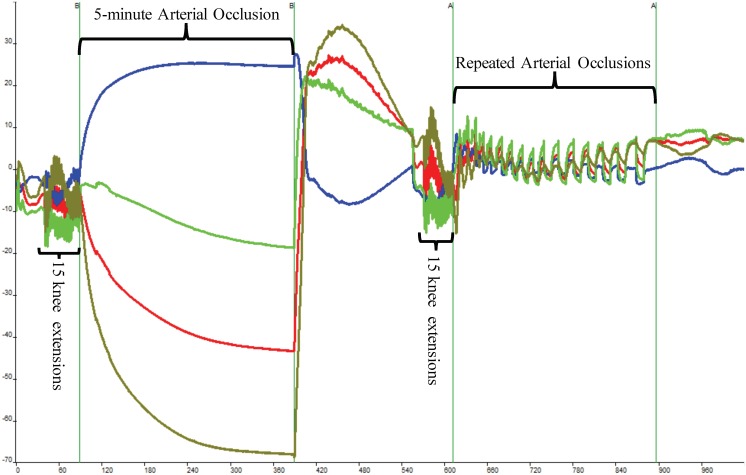
Depiction of raw near-infrared spectroscopy (NIRS) data during a single Muscle Oxidative Capacity (MOC) test. Fifteen isokinetic knee extensions were used to increase skeletal muscle metabolic rate which was followed by a 5-min occlusion induced by a high-pressure cuff. Following a 3-min recovery period, another set of 15 isokinetic knee extensions followed by a series of short arterial conclusions were performed. The blue line indicates deoxyhemoglobin (HHb), red indicates oxyhemoglobin, and green line depicts total hemoglobin.

The subject sat still in the Biodex System Pro 4 chair for 60 s, after which the pneumatic cuff was inflated and deflated for 30 s. A series of three 10-s occlusions occurred. After the recovery period, the subject completed 15 IK knee extensions on the Biodex at an angular rate of 300°/s for the concentric and eccentric motions. After the 15th extension, the subject’s right leg was extended to 120°, locked in place using the Biodex leg extension attachment, and the cuff was inflated. The occlusion lasted for 5 min. Once the cuff was released, the subject recovered for 3 min without moving as shown in **Figure 2**. This protocol served as the 100% and 0% oxygenation HHb levels for the muscle oxidative capacity calculations (Ryan et al., 2012).

Following another 3-min recovery period, the subject performed another set of 15 IK knee extensions at 300°/s. Immediately after the 15th extension, the subject’s right leg was extended to approximately 120° and repeated short arterial occlusions were performed. These were used to assess changes in metabolic rate (Ryan et al., 2013). The initial occlusions were 5 s in length followed by 5 s of rest (occlusions 1 through 5). The subsequent occlusions were 7 s in length followed by 7 s of rest (occlusions 6 through 10) and 10 s in length and 10 s of rest (occlusions 11 through 15) as shown in **Figure 2**. The PortaMon NIRS signals were corrected and used to calculate the subject’s muscle oxidative capacity time constant (Tc) and rate constant (*k*) using MatLab (Mathworks, Natick, MA, United States) as outline in Ryan et al. (2012, 2013).

#### Data Analysis

The SPSS Faculty Pack (version 23, IBM, ON, Canada) was used for all statistical analyses with the level of significance set at *p* < 0.05. Data were reported in means ± SEM. For the MHE analysis, a two-way repeated-measures analysis of variance (ANOVA) model (2 × 3) on pre-/post-WI on WI 1, 3, and 5 for the dive week was used. For the muscle oxidative capacity analysis, a two-way repeated-measures ANOVA model (2 × 5) on pre-/post-WI on WI 1–5 for the dive week was used. MHE and MOC recovery periods were analyzed via a one-way repeated-measures ANOVA model (1 × 3) on pre-WI 1 (baseline), 24-h post-WI, and 72-h post-WI. Conversely, for 50-repetition maximal isokinetic knee extension analysis, a two-way repeated-measures ANOVA model (2 × 2) on pre-/post-WI on WI 1 and 3 were used. Recovery analysis used a one-way repeated-measures ANOVA model (1 × 3) on pre-WI 1 (baseline), pre-WI 5, and 24-h post-WI. When appropriate, Bonferroni correction was used to adjust the alpha when *post hoc* tests were performed.

## Results

### MHE

**FIGURE 3:**
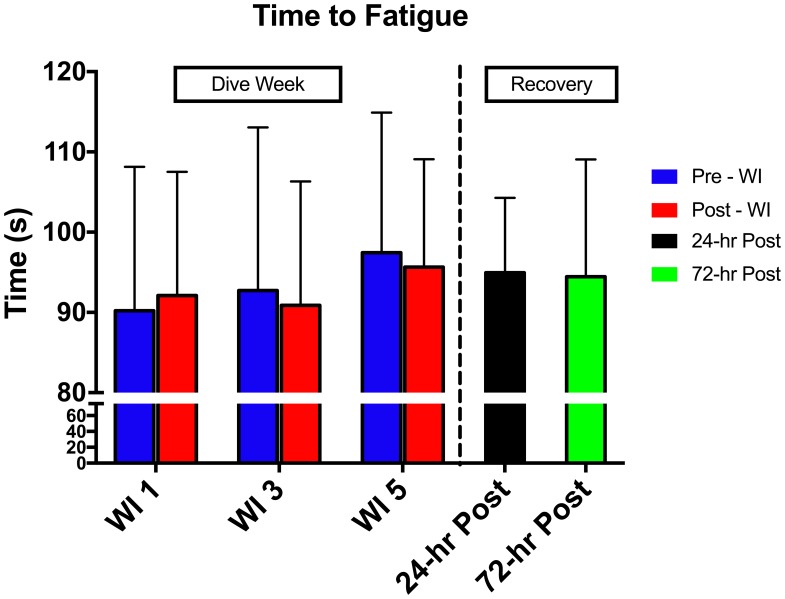
Forty percent MHE time-to-fatigue results. Across subject (*n* = 15) mean ± SEM is shown. No change occurred in the MHE time-to-fatigue testing.

As shown in **Figure 3**, no main effects occurred with time-to-fatigue. In **Table 3**, total normalized amplitude exhibited a 4.8 and 7.2% increase on WIs 3 and 5 (*p* = 0.041) and returned to baseline by 24-h post-WI. No other changes arose with the SEMG metrics.

**FIGURE 4:**
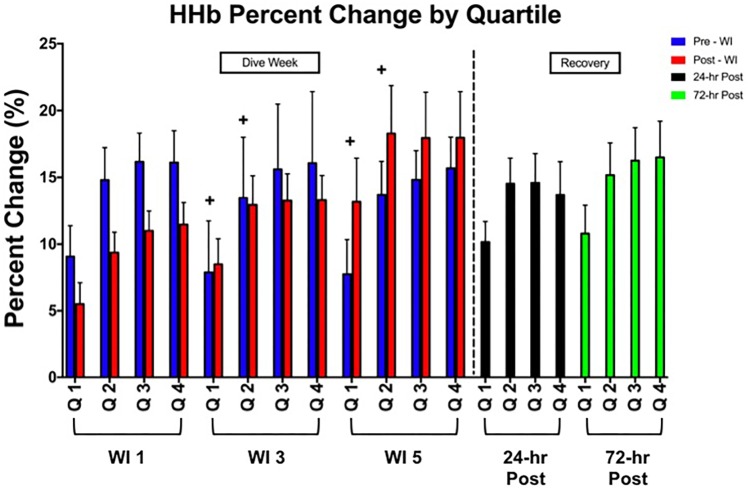
Forty percent MHE HHb percent change by quartile results. Across subject (*n* = 15) mean ± SEM is shown. “ + ” Significant day main effect *p* < 0.05 versus WI 1. Each quartile WI quartile was compared to its baseline counterpart. For example, WI 3 Q1 compared to WI 1 Q1; WI 3 Q2 compared to WI 1 Q2; WI 5 Q1 compared to WI 1 Q1; WI 5 Q2 compared to WI 1 Q2.

No main effects were observed in total HHb or with all TSI metrics as shown in **Table 3** as compared to WI 1. The first quartile of HHb concentration increased by 6 and 35% increase on WIs 3 and 5 (*p* = 0.026). Furthermore, as shown in **Figure 4**, the second quartile increased by 9 and 32% on WIs 3 and 5 (*p* = 0.049).

### Muscle Oxidative Capacity

A 24.9% (*p* = 0.044) increase in the muscle oxidative capacity rate constant (*k*) occurred on WI 4. Aside from this, no other changes in T_c_ were seen during the dive week and the recovery periods.

### 50-Repetition Maximal Isokinetic Knee Extension

**FIGURE 5:**
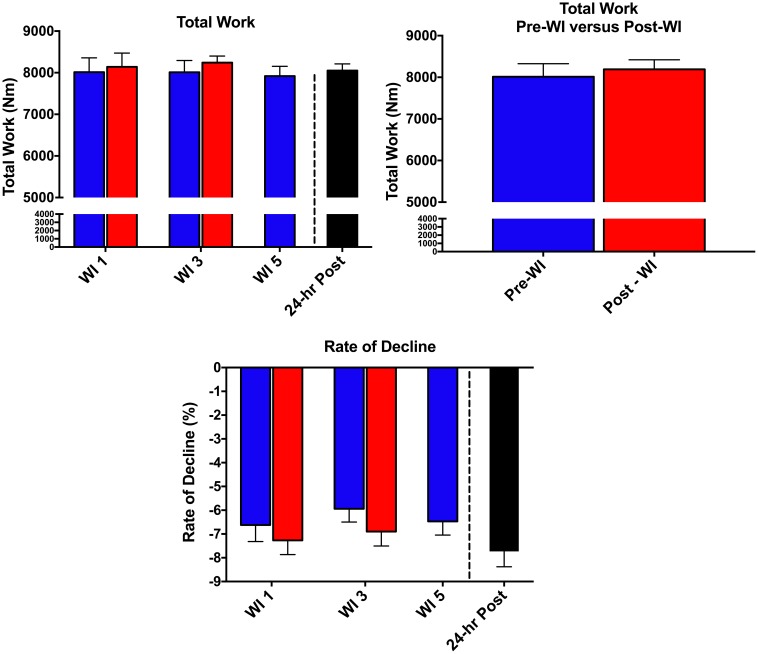
Fifty-repetition maximal isokinetic knee extension results. Across subject (*n* = 15) mean ± SEM is shown. No change occurred during the 50-repetition maximal isokinetic knee extension.

As shown in **Figure 5**, there was no change in total work neither pre- nor post-WI on days 1 and 3. Additionally, there was no change during the dive week or recovery. Similarly, the rate of fatigue did not change across all testing sessions. Repetitions 2–4 were compared to baseline. No changes were seen in these measurements. Additionally, all subjects had noticeable negative slopes changes of force output throughout the 50-repetitions, indicating maximal exertion was provided for each testing session. Furthermore, no change occurred between pre- and post-WI and dive week peak HHb concentrations and peak TSI.

As shown in **Table 2**, steady-state HHb results post-WI 3 showed a 17% increase in steady-state HHb as compared to pre-WI 3 (*p* = 0.036); however, steady-state HHb did not experience a day effect (**Table 2**). Steady-state TSI had a 4.5% decrease on WI 3 and 5 (*p* = 0.002) and continued with a 4% reduction on 24-h post-WI (*p* = 0.028). As demonstrated by **Table 2**, no pre-/post-WI main effect was seen with steady state TSI. The HHb and TSI steady-state results are associated with low effect sizes denoting the small probability of seeing this change within the population (Cohen, 1988).

**Table 2:** 50-repetition isokinetic knee extension NIRS quartile results.

Variable	Time					*p*-value			
	Dive day			Recovery		Main effect		Interaction	Main effect
	Day 1	Day 3	Day 5	24-hr Post	72-hr Post	Pre/Post	Day	Pre/Post x Day	Recovery
**50-Repetition isokinetic knee extension**									
**HHb peak balue (%)**									
Pre	14.37 ± 2.33	14.59 ± 1.57	16.33 ± 1.68	18.08 ± 2.34	–	0.101	0.33	0.101	0.068
Post	14.36 ± 2.32	16.48 ± 1.92	–	–	–				
**HHb steady state value (%)**									
Pre	12.61 ± 1.94	12.93 ± 1.32	14.63 ± 1.42	16.14 ± 2.14	–	**0.036^∗^**	0.109	0.314	0.077
Post	12.84 ± 1.65	15.13 ± 1.79	–	–	–				
**TSI peak value (%)**									
Pre	57.61 ± 1.59	54.43 ± 2.13	56.11 ± 1.24	55.28 ± 1.08	–	0.354	0.075	0.523	0.149
Post	57.92 ± 1.23	56.28 ± 1.26	–	–	–				
**TSI steady state value (%)**									
Pre	61.78 ± 1.08	58.01 ± 1.30	58.72 ± 1.25	58.67 ± 0.87	–	0.941	**0.002^∗^**	0.177	0.028
Post	60.72 ± 1.17	58.93 ± 1.05	–	–	–				

*All results (n = 15) reported as mean ± SEM. Bold indicates statistical significance (p < 0.05) with effect size ≥0.70. “^∗^” denotes low effect size, effect size ≤0.20.*

## Discussion

By definition, muscular endurance is defined as the ability for a muscle to perform the same workload repeatedly (Powers and Howley, 2011). In contrast to our hypothesis, resting, consecutive, long-duration WIs did not alter skeletal neuromuscular endurance performance. No change occurred in the performance measures for the 50-repetition maximal isokinetic knee extension and MHE. As such, these results disprove the hypothesis put forth. To our knowledge, this study is the first to analyze neuromuscular activation and hemoglobin dynamics in skeletal muscle fatiguing endurance protocols after consecutive, resting, long duration WIs at 1.35 ATA. The endurance performance characteristics of load bearing versus non-load bearing limbs are quite interesting and unexpected.

### 40% Maximal Handgrip Endurance Performance

Subject performance during this static, endurance exercise was not affected by the WIs. The MHE result matches the results from previous consecutive, resting, long-duration WI testing (Florian et al., 2014). Total normalized SEMG results illustrated increased brachioradialis activation throughout the dive week. As shown in **Table 3**, the increase in total normalized amplitude suggests that more brachioradialis muscle fiber activation was required to perform the same amount of work on WIs 3 and 5 as compared to WI 1 (*p* = 0.041). A comparable response was seen with the Kamiya et al. and Fu et al. studies. Both studies experience increased muscle sympathetic nerve activity post-HBDR and post-space flight. The difference occurred in endurance performance. Fu et al. (2002) showed a 32% reduction in time-to-fatigue post-space flight. Our study, Florian et al., and Kamiya et al. studies did not see a change in MHE time-to-fatigue. The increased neuromuscular amplitude and muscle sympathetic nerve activity show that the forearm flexors need to work harder to maintain performance. Altogether, these studies show that the neuromuscular activation is greater in order to preserve performance after extensive exposures to microgravity environments. One aspect is for certain, the data from this study suggest five consecutive long-duration WIs may not be enough exposure time in a microgravity environment to cause a decrease to forearm flexor endurance performance.

**Table 3:** 40% MHE SEMG and NIRS quartile results.

Variable	Time					*p*-value			
	Dive Day			Recovery		Main effect		Interaction	Main effect
	Day 1	Day 3	Day 5	24-hr Post	72-hr Post	Pre/Post	Day	Pre/Post x Day	Recovery
**40% MHG endurance test**									
**SEMG, normalized RMS (%)**									
Pre	100 ± 0	109 ± 9	105 ± 10	105 ± 13	120 ± 14	0.659	**0.041**	0.548	0.731
Post	106 ± 8	108 ± 9	116 ± 13	–	–				
**SEMG, normalized RMS (%) – Quartile 1**									
Pre	100 ± 0	152 ± 36	146 ± 28	140 ± 35	137 ± 21	0.241	0.245	0.505	0.285
Post	129 ± 21	127 ± 23	174 ± 51	–	–				
**SEMG, normalized RMS (%) – Quartile 2**									
Pre	100 ± 0	169 ± 43	130 ± 20	132 ± 29	148 ± 25	0.292	0.231	0.222	0.287
Post	126 ± 17	141 ± 31	154 ± 35	–	–				
**SEMG, normalized RMS (%) – Quartile 3**									
Pre	100 ± 0	152 ± 25	129 ± 18	119 ± 15	145 ± 21	0.272	0.135	0.243	0.11
Post	119 ± 16	128 ± 16	137 ± 25	–	–				
**SEMG, normalized RMS (%) – Quartile 4**									
Pre	100 ± 0	117 ± 10	97 ± 8	99 ± 7	114 ± 14	0.345	0.14	0.091	0.514
Post	107 ± 8	105 ± 11	107 ± 9	–	–				
**TSI**									
Pre	62.95 ± 3.35	59.39 ± 1.82	60.55 ± 2.73	60.73 ± 2.24	61.16 ± 2.26	0.342	0.158	0.248	0.787
Post	58.9 ± 1.3	59.63 ± 1.32	60.75 ± 2.15	–	–				
**TSI (%) – Quartile 1**									
Pre	64.85 ± 3.15	61.84 ± 1.81	62.84 ± 2.26	62.29 ± 1.33	62.81 ± 2.35	0.171	0.78	0.457	0.684
Post	60.46 ± 1.19	61.1 ± 1.2	61.49 ± 1.59	–	–				
**TSI (%) – Quartile 2**									
Pre	62.56 ± 3.27	58.39 ± 1.92	59.18 ± 2.73	59.57 ± 2.89	60.08 ± 2.86	0.542	0.564	0.321	0.676
Post	58.72 ± 1.32	59.3 ± 1.5	58.99 ± 1.87	–	–				
**TSI (%) – Quartile 3**									
Pre	61.93 ± 3.15	57.82 ± 1.82	58.8 ± 2.75	59.28 ± 2.35	60.53 ± 2.43	0.915	0.728	0.134	0.825
Post	58.17 ± 1.48	59.57 ± 1.27	61.38 ± 2.37	–	–				
**TSI (%) – Quartile 4**									
Pre	61.71 ± 3.08	58.93 ± 1.58	61.26 ± 2.62	60.74 ± 2.13	61.19 ± 1.72	0.904	0.341	0.397	0.876
Post	58.9 ± 1.2	59.54 ± 0.87	62.81 ± 2.25	–	–				

*All results (n = 15) reported as mean ± SEM. Bold indicates statistical significance (p < 0.05) with effect size ≥ 0.70.*

Another plausible influence for the total SEMG amplitude increase shown in **Table 3** is the forearm flexor fiber type composition. Type II fibers fatigue quicker than type I (Powers and Howley, 2011). The more type II fibers, the greater the SEMG amplitude in order to maintain the muscular endurance performance. The primary technique to determine muscle fiber composition is through invasive fiber typing techniques (Powers and Howley, 2011). The may be an aspect to explore in future studies.

Forearm flexor pre-WI and post-WI first and second quartile HHb concentrations were greater on WI 3 (Q1: *p* = 0.009, 4.4% increase; Q2: *p* = 0.036, 4.7% increase) and WI 5 (Q1: *p* = 0.022, 5.6% increase; Q2: *p* = 0.04, 5.5% increase) as compared to WI 1 quartiles (**Figure 5**). This result shows a slight decrease in oxygen availability occurs in the local tissue during the first two time-quartiles of the MHE (Tanaka et al., 2016). This oxygen debt appears not to effect handgrip endurance performance as time-to-fatigue did not change. Yet, these data show—albeit initially—that either less oxygen was available for consumption or more oxygen was used (Ryan et al., 2012, 2013; Tanaka et al., 2016).

During the initial seconds and minutes of steady state, submaximal endurance exercise (i.e., running and cycling), a sharp increase in HHb concentration arises (i.e., lower oxygen availability). The target muscle stays in a steady hypoxic state during the exercise once it reaches the voluntary rate of exertion (Ufland, 2012; Hopker et al., 2016). It is not until near fatigue that the HHb concentrations start to rise sharply (Hopker et al., 2016). This characteristic was not seen during the MHE. In **Figure 4**, the HHb concentrations rise through the duration of the handgrip exercise with the HHb concentration peaking during the forth quartile (i.e., at the point of exhaustion). However, no change in TSI occurred. This suggests that oxygen availability might not have been an issue. This outcome may be the result of the MHE not constituting a truly aerobic endurance protocol. As shown in **Table 3**, all time to exhaustion results were under 2 min. This result coincides with the acceptable length of time for the anaerobic energy system (Powers and Howley, 2011). Given that time to exhaustion is unaffected by the WIs, and taking into considering that the flexor carpi radialis is under hypoxic conditions while performing an anaerobic exercise, oxygen availability is not the limiting factor. This leads to a possible conclusion that stored adenosine-triphosphate availability is unaffected by consecutive, long-duration WIs during shorter duration muscular endurance exercises (Powers and Howley, 2011).

### Muscle Oxidative Capacity

The increase in the rate constant (*k*) on WI 4 may signify an increase in muscle oxidative capacity. However, no change is seen on WIs 3 and 5. For this change on WI 4 to be physiologically significant, we would expect changes in k on WIs 3 and 5. Additionally, changes in the Tc would occur. The variables *k* and Tc are mathematical inverses of each other. For a true change to exist, changes in both variables would need to occur at the same time point. This did not happen in this case. Due to this, the significance found in the variable *k* on WI 4 is not considered physiologically significant. Further investigation is necessary to confirm this result and interpret its physiological significance.

### 50-Repetition Knee Extension Endurance Performance

The quadriceps muscular endurance did not change throughout the dive week. Total work and fatigue indices were unaffected by the consecutive, resting, long-duration WIs. In addition, localized tissue oxygenation was unaffected. This result illustrates the difference between maximal and submaximal exertion that occurs with quadriceps performance when exposed to consecutive, long-duration WIs. Previously, we reported an 11% and 5% increase post-WI than pre-WI maximal isokinetic exercise on WIs 3 and 5 (Myers et al., 2017). Furthermore, overall performance increased by 3% throughout the dive week. The recovery during maximal isokinetic exertion and no change to isokinetic endurance exertions demonstrate the resiliency of the quadriceps. These performance characteristics demonstrate how this load-bearing muscle group reacts and adapts to the WIs unloading environment.

The quadriceps and forearm flexor endurance performance illustrate how limb differences do not exist under short-term unloading conditions, such as those produced by long-duration WIs. As previously reported, skeletal muscle performance differs between load and non-load bearing limbs during maximal efforts. These results demonstrate that skeletal muscle endurance for load-bearing and non-load bearing limbs are preserved under unloading conditions (Pastacaldi et al., 2004). The protective mechanisms themselves are not fully understood. Perhaps, one explanation may in fact be tissue oxygenation.

In this study, we measured tissue oxygenation via changes in HHb concentrations for load bearing and non-load bearing muscles. HHb concentrations and TSI measurements of the VL were unchanged. A similar result occurred with flexor carpi radialis, especially with the third and fourth quartile measurements (**Figure 4**). These results demonstrate that oxygen offloading was unaffected by the consecutive WIs. Since TSI is an absolute measure of O_2_Hb and HHb is the measure of deoxygenation, these two measures demonstrate the oxygen extraction activity of the local muscle (Kawahara et al., 2008; Tanaka et al., 2016). Under normal conditions, typical skeletal muscle saturation ranges between 60 and 80%. These metrics indicate that the constant flow of oxygen to the working muscle was not affected by the WIs, and therefore was not a limiting factor to performance (Kawahara et al., 2008; Powers and Howley, 2011). This insight shows how skeletal muscle endurance performance can be preserved during the multiple, short-term unloading exposures of the WIs.

These results parallel what is seen in long-duration unloading analogs. The study by Weber et al. showed that soleus tissue oxygenation index was unchanged after 8 weeks of unloading (Kawahara et al., 2008; Weber et al., 2014). Our TSI measurements yielded the same result. The Weber et al. study, along with our study, demonstrates the ways in which oxygen offloading kinetics remain unchanged in acute and chronic unloading situations. The totality of these short and long-term results suggests that hemoglobin function and oxygen uptake by the muscle are uninhibited while exposed to an unloading environment.

### Experimental Considerations

This study is the first to consider the effects of consecutive, resting, long-duration WIs on skeletal muscle endurance, neuromuscular activation, and HHb concentrations. Nevertheless, special consideration must occur when interpreting the results of this study. At the outset, the exercise protocols occurred 1–1.5 h after WI. If changes in oxygen offloading kinetics occurred prior to protocol initiation, these changes could not be detected. Additionally, the HHb concentrations were not normalized to ischemic or hyperemic responses (Ryan et al., 2012, 2013). If we were to follow typical ischemic protocols on the forearm and quadriceps during the entire dive week, we would have caused undue stress and discomfort to the subjects (Kawahara et al., 2008; Ryan et al., 2012, 2013). Furthermore, disagreement exists on how to normalize raw NIRS data in the absence of the ischemic protocol (Ufland et al., 2012). It is unclear if this change in procedure would change the results.

The conclusions drawn from the 50-repetition maximal isokinetic knee extension test are limited. The performance data is limited in explaining the main effects. It is important to note that post-WI 5 testing did not occur due to interference with other exercise protocols. Specifically, the 50-repetition maximal isokinetic knee extension protocol would have caused undue fatigue and confounded the results of the other testing. In looking at **Figure 3**, the rate of fatigue for pre-WI 5 was greater than the previous same time points. It is unknown if the rate of fatigue would be greater at post-WI 5. Without this data point, we are unable to draw a conclusion if performance changed at post-WI 5.

## Conclusion

Our findings demonstrate that our resting, consecutive, long-duration WIs did not cause changes in muscular endurance and oxygen kinetics. Furthermore, there were no limb differences present at submaximal endurance exertions. It appears as if oxygen offloading kinetics may be independent of muscle endurance performance, and short-term unloading does not affect performance. Further research is necessary to understand the physiological mechanisms for the resilient sustainment of skeletal muscle endurance performance under these conditions.

## Ethics Statement

This study was carried out in accordance with the recommendations of Navy Experimental Dive Unit and Florida State University Institutional Review Boards. The protocol was approved by the Navy Experimental Dive Unit and Florida State University Institutional Review Boards. All subjects gave written informed consent in accordance with the Declaration of Helsinki.

## Author Contributions

CM is the primary author. Furthermore, he performed the data collection and data analysis for this project. J-SK and JF performed the data analysis and interpretation. Both provided mentorship for the structure, planning, and execution for this project. MM wrote the MatLab analysis program to analyze the near infrared spectroscopy data (NIRS) used for this project. Additionally, she assisted CM with the data collection for this project. KM provided data collection guidance and analysis for MOC data.

## Conflict of Interest Statement

The authors declare that the research was conducted in the absence of any commercial or financial relationships that could be construed as a potential conflict of interest.

## Abbreviations

ATA: atmospheres absolute
HHb: Deoxygenated hemoglobin
IK: isokinetic
MHE: maximum handgrip endurance
MHG: maximal handgrip
MVIC: maximal voluntary isometric contraction
NIRS: near infrared spectroscopy
ANOVA: analysis of variance
SEMG: surface electromyography
TSI: tissue saturation index
VL: vastus lateralis
WI: water immersion
